# Prevalence of the Risk Factors of Delirium Among COVID-19 Patients in the Intensive Care Unit at King Abdullah Medical City, Makkah: A Single-Center Study

**DOI:** 10.7759/cureus.31032

**Published:** 2022-11-02

**Authors:** Yasmine Demiroz, Omniyyah Tukruni, Nada Almalayo, Alaa Ahmed, Amjad Alzahrani, Rinad Aljohani, Nouf Alzahrani, Haneen Albeeshi

**Affiliations:** 1 Psychiatry, King Abdullah Medical City, Makkah, SAU; 2 Medical Student, Umm Al-Qura University, Makkah, SAU; 3 Medical Student, Taibah University, Medina, SAU; 4 Medical Student, Batterjee Medical College, Jeddah, SAU

**Keywords:** sars-cov-2, icu, mortality, covid-19, delirium

## Abstract

Background

Coronavirus disease 2019 (COVID-19) patients admitted to the intensive care unit (ICU) are at a higher risk of developing delirium. In this study, we estimated the incidence of delirium and its risk factors in ICU patients with COVID-19 at King Abdullah Medical City (KAMC), Makkah, Saudi Arabia.

Methodology

We conducted a retrospective, analytical, cohort study of adult COVID-19 patients admitted to the ICU of KAMC between May 2020 and July 2021. Data were collected from electronic medical records.

Results

Of the 406 examined patients with COVID-19 aged >18 years, 55 developed delirium in the ICU setting. The incidence rate was 0.59% per 100 ICU days in these 55 patients; the mean age was 62.36 ± 17.9 years, and 65.5% were men. Binary logistic regression showed that age (p = 0.027), nationality (p = 0.045), presence of infectious diseases other than COVID-19 (p = 0.047), and ICU outcome (p = 0.013) were significant risk factors for developing delirium. The clinical presentation and prognosis of patients who developed delirium were assessed using the Acute Physiology and Chronic Health Evaluation II and Sequential Organ Failure Assessment scores, and the mean scores were 16.13 ± 7.96 and 5.25 ± 3.48, respectively. The mean length of ICU stay was 22.2 ± 33.3 days; 39 (70.9%) patients were discharged and 16 (29.1%) died.

Conclusions

Older age, nationality, infections, and ICU outcomes were risk factors for developing delirium in hospitalized COVID-19 patients at KAMC. Early detection of cognitive comorbidities and delirium in these patients is important.

## Introduction

In December 2019, several patients in Wuhan, China, experienced pneumonia of unknown cause. This atypical infection was soon discovered to be caused by the severe acute respiratory syndrome coronavirus 2 (SARS-CoV-2). Later, the respiratory disease caused by this virus was termed coronavirus disease 2019 (COVID-19). The World Health Organization (WHO) designated it an international public health emergency in January 2020 and declared it a pandemic in March 2020 in response to the progressive increase in infection and mortality rate [1,2]. Owing to the scientists’ contributions to the literature, COVID-19 is well recognized by its respiratory signs and symptoms and the diagnostic criteria [3]. Although the clinical manifestations of COVID-19 are well known, multiple dilemmas remain in question as several medical conditions have been shown to be associated with COVID-19 infection.

Since the start of the pandemic, the number of patients requiring treatment in intensive care units (ICUs) worldwide has increased [4]. According to the Saudi Ministry of Health Statistics, the total number of critical cases reached approximately 1,424 out of 10,788 active cases in July 2021 [5]. Clinicians have observed that several patients hospitalized for acute respiratory distress syndrome (ARDS) may develop neurological symptoms, including delirium. The evidence is established in the literature, for example, in France, some ARDS cases caused by SARS-CoV-2 infection have reportedly been associated with encephalopathy, agitation, and confusion, as well as corticospinal tract symptoms in an observational series [6]. Additionally, a recent review of neuropsychiatric manifestations following COVID-19 infection suggested a high frequency of delirium [7].

According to the Diagnostic and Statistical Manual of Mental Disorders, fifth edition (DSM-5), delirium is defined as “a disturbance of attention or awareness that is accompanied by a change in baseline cognition that cannot be better explained by a preexisting or evolving neurocognitive disorder” [8]. Delirium is associated with significant mortality and morbidity in patients with COVID-19 [9]. The risk factors for delirium are related to both predisposing (patient-related) and precipitating (hospital-related) risk factors [10], such as admission to the ICU, length of hospital stay, alcoholism, and advanced age [11]. Additionally, inflammation and neuroinflammation in COVID-19 patients are associated with an increased risk of delirium and social isolation from family members [12]. In fact, the preventive measures implemented by health authorities, such as social distancing, may account for a portion of the issue as COVID-19 patients in ICUs are isolated from their families, thus losing their orientation to the time of the day. Despite all risk factors and a high incidence of delirium in ICU patients with COVID-19, a gap in the literature lies in investigating this topic, especially in Saudi Arabia, where little attention has been paid to the clinical characteristics and outcomes of ICU patients with delirium [10-12]. Therefore, this study aimed to investigate the development of delirium and its risk factors in ICU patients with COVID-19 at King Abdullah Medical City (KAMC) to provide further knowledge to minimize and prevent the occurrence of delirium and its unfavorable outcomes in COVID-19 ICU patients.

## Materials and methods

Study population

A retrospective, analytical, cohort study was conducted in the ICU Department at KAMC, Makkah. The study included all patients infected with COVID-19 who were diagnosed with delirium, based on the medical records of the primary team, during their stay in the ICU between March 2020 and July 2021.

The researcher was committed to all ethical considerations required to conduct the research. The Institutional Review Board (IRB) committee at KAMC provided ethical approval to conduct this study. Verbal consent was obtained from patients, participants’ privacy was maintained, and the responses remained confidential. This study followed the tenets of the Declaration of Helsinki. Data were collected between August 11, 2021, and September 14, 2021, using population data from medical records.

Sampling technique

A constructed data sheet was used to collect a population sample from the KAMC database based on the inclusion criteria. The inclusion criteria were the existence of delirium (yes/no), all patients aged ≥18 years (because KAMC admits adult patients only) who were infected with COVID-19 and had delirium in the ICU setting, patients admitted to the ICU with severe ARDS, and those diagnosed with COVID-19, which was defined by a positive polymerase chain reaction test for SARS-CoV-2 on nasopharyngeal swabs. However, patients with neurocognitive disorders were excluded because the treating physician may misdiagnose delirium and confuse it with other diagnoses such as dementia or other neurocognitive disorders.

Study tool

A structured and constructed data sheet was developed based on the reviewed literature. The data sheet was used to collect patient data, such as the patient’s serial code and initials. Patient medical record data were recorded, for example, age at ICU admission, sex, nationality, height, weight, body mass index (BMI), comorbidities, and risk factors, such as cardiac diseases (myocardial infarction and congestive heart failure), chronic pulmonary diseases (chronic obstructive pulmonary disease, asthma), infections (other than COVID-19), renal impairment diseases, cancer (leukemia, lymphoma, solid cancer), diabetes, and hypertension. Moreover, the date of delirium diagnosis was recorded to calculate the duration of the disease course.

ICU data were determined using the calculated Acute Physiology and Chronic Health Evaluation II (APACHE II) score, Sequential Organ FailureAssessment (SOFA) score, treatment/procedure used in the ICU setting, use of supplemental oxygen, ICU outcome (death or discharge), and date of ICU outcome (death or discharge). The severity score and mortality estimate technique known as APACHE II was created using data from a substantial sample of ICU patients in the United States. A broad assessment of the severity of a condition is provided using a point score based on the beginning values of 12 common physiologic parameters, age, and past health status. Furthermore, the Sequential Organ Failure Assessment (SOFA) score, which can assess individual or aggregate organ dysfunction, is a concise and objective score that allows for the calculation of the number and severity of organ dysfunction in six different organ systems, namely, respiratory, coagulatory, liver, cardiovascular, renal, and neurologic system.

Statistical analysis

SPSS version 25 (IBM Corp., Armonk, NY, USA) was used for data analysis. Discrete variables were reported using counts and percentages, whereas continuous variables were reported as means and standard deviations. Comparative analysis was performed between patients who developed delirium and those who did not using the Mann-Whitney U test for continuous variables and the chi-square test or Fisher’s exact test for categorical variables. Binary logistic regression analysis was used to determine predictors of developing delirium.

Ethical approval

Ethical approval was obtained on August 10, 2021, from the KAMC ethical committee. Confidentiality was maintained by coding the patients’ medical record numbers (MRNs) in a separate MRN coding sheet. Information defining the patient’s identity was collected, protected, and made inaccessible. No personal contact information was collected, patient data were kept strictly confidential, and only the research team had access to this data.

## Results

The total number of ICU patients infected with COVID-19 was 406, of whom 55 developed delirium. The mean age of the total sample was 58.51 ± 16.23 years; 62.36 ± 17.92 years for patients with delirium, and 57.90 ± 15.89 years for patients without delirium. Of these patients, 245 (60.3%) were men and 161 (39.7%) were women. Of the patients who developed delirium, 36 (65.5%) were men. Most patients who developed delirium were of Saudi nationality; 48 of the 55 (87.3%) patients who developed delirium were of Saudi origin, and the remaining 266 (75.8%) did not develop delirium. The mean height of patients with delirium was 162.82 ± 13.19 cm, which was lower than that of patients without delirium (163.51 ± 9.13 cm). The mean weight of patients with delirium was 82.12 ± 25.43 kg, which was higher than that of patients without delirium (79.55 ± 18.30 kg). Of the patients who developed delirium, only nine (16.4%) had cardiac disease compared to 74 (21.1%) without delirium. Other risk factors included chronic pulmonary disease in 37 (10.5%) patients without delirium and in seven (12.7%) patients with delirium, renal disease in 67 (19.1%) patients without delirium and in 11 (20%) patients with delirium, and cancer in 42 (12%) patients without delirium and in nine (16.4%) patients with delirium. Diabetes and hypertension patients in the non-delirium group and the delirium group comprised 199 (56.7%) and 208 (59.3%) and 28 (50.9%) and 33 (60%) patients, respectively. Clinical presentation and prognosis were assessed using APACHE II and SOFA scores. The mean APACHE II score in COVID-19 patients who did and did not develop delirium was 16.13 ± 7.96 and 15.11 ± 7.65, respectively. The mean SOFA score in COVID-19 patients who did and did not develop delirium was 5.25 ± 3.48 and 5.26 ± 3.75, respectively. The ICU outcomes were as follows: 215 (61.3%) COVID-19 patients who did not develop delirium and 39 (70.9%) COVID-19 patients who developed delirium were discharged; 136 (38.7%) COVID-19 patients who did not develop delirium and 16 (29.1%) COVID-19 patients who developed delirium passed away (Table 1).

**Table 1:** The demographic characteristics of patients with delirium and those without delirium. P-values in bold show that the variables are statistically significant. SD: standard deviation; APACHE II: Acute Physiology and Chronic Health Evaluation II; SOFA: Sequential Organ Failure Assessment; DM: diabetes mellitus; HTN: hypertension; ICU: intensive care unit

Variable	All patients (n = 406) 100%	Patients without delirium (n = 351) 86.5%	Patients with delirium (n = 55) 13.5%	P-value
Age				0.046
Mean ± SD	58.51 ± 16.23	57.90 ± 15.89	62.36 ± 17.92	
Median (minimum-maximum)	60.50 (20-98)	60 (20-98)	68 (25-91)	
Gender				0.405
Female	161 (39.7%)	142 (40.5%)	19 (34.5%)	
Male	245 (60.3%)	209 (59.5%)	36 (65.5%)	
Nationality				0.058
Non-Saudi	92 (22.7%)	85 (24.2%)	7 (12.7%)	
Saudi	314 (77.3%)	266 (75.8%)	48 (87.3%)	
Height				0.737
Mean ± SD	163.41 ± 9.76	163.51 ± 9.13	162.82 ± 13.19	
Median (minimum-maximum)	165 (91-200)	165 (130-200)	165 (91-180)	
Weight				0.908
Mean ± SD	79.90 ± 19.40	79.55 ± 18.30	82.12 ± 25.43	
Median (minimum-maximum)	79.60 (30-188)	80 (30-180)	76 (46-188)	
Cardiac disease				0.420
No	323 (79.6%)	277 (78.9%)	46 (83.6%)	
Yes	83 (20.4%)	74 (21.1%)	9 (16.4%)	
Chronic pulmonary disease				0.628
No	362 (89.2%)	314 (89.5%)	48 (87.3%)	
Yes	44 (10.8%)	37 (10.5%)	7 (12.7%)	
Infections				0.292
No	290 (71.4%)	254 (72.4%)	36 (65.5%)	
Yes	116 (28.6%)	97 (27.6%)	19 (34.5%)	
Renal disease				0.873
No	328 (80.8%)	284 (80.9%)	44 (80%)	
Yes	78 (19.2%)	67 (19.1%)	11 (20%)	
Cancer				0.360
No	355 (87.4%)	309 (88%)	46 (83.6%)	
Yes	51 (12.6%)	42 (12%)	9 (16.4%)	
APACHE II score				0.368
Mean ± SD	15.25 ± 7.69	15.11 ± 7.65	16.13 ± 7.96	
Median (minimum-maximum)	14 (2-86)	14 (2-86)	14 (5-56)	
SOFA score				0.821
Mean ± SD	5.26 ± 3.71	5.26 ± 3.75	5.25 ± 3.48	
Median (minimum-maximum)	4 (0-24)	4 (0-24)	4 (0-14)	
DM				0.422
No	179 (44.1%)	152 (43.3%)	27 (49.1%)	
Yes	227 (55.9%)	199 (56.7%)	28 (50.9%)	
HTN				0.917
No	165 (40.6%)	143 (40.7%)	22 (40%)	
Yes	241 (59.4%)	208 (59.3%)	33 (60%)	
ICU outcomes				0.169
Discharged	254 (62.6%)	215 (61.3%)	39 (70.9%)	
Died	152 (37.4%)	136 (38.7%)	16 (29.1%)	

Delirium was significantly associated with age 68 years versus 60 years (p = 0.027). Significantly, the prevalence of comorbidities and risk factors associated with delirium were infections other than COVID-19, 97 (27.6%) versus 19 (34.5%) (p = 0.047) when compared to patients without delirium (Table 1). We used binary logistic regression analyses, including all variables with delirium, and found that age, nationality, infections other than COVID-19, and ICU outcomes were significant risk factors for delirium (Table 2).

**Table 2:** Binary logistic regression analyses. P-values in bold show that the variables are statistically significant at α = 0.05. CI: confidence interval; OR: odds ratio; APACHE II: Acute Physiology and Chronic Health Evaluation II; SOFA: Sequential Organ Failure Assessment; DM: diabetes mellitus; HTN: hypertension; ICU: intensive care unit

Variable	OR (95% CI)			P-value
	Exp (B)	Lower	Upper	
Age	1.025	1.003	1.047	0.027
Gender	0.593	0.287	1.226	0.159
Nationality	0.408	0.170	0.979	0.045
Height	0.979	0.948	1.012	0.206
Weight	1.008	0.993	1.022	0.308
Cardiac disease	1.392	0.619	3.131	0.424
Chronic pulmonary disease	0.793	0.323	1.948	0.613
Infections	0.487	0.239	0.992	0.047
Renal disease	0.917	0.418	2.011	0.828
Cancer	0.653	0.278	1.536	0.329
APACHE II score	1.010	0.966	1.058	0.653
SOFA score	1.003	0.898	1.120	0.962
DM	1.676	0.825	3.407	0.154
HTN	1.002	0.480	2.090	0.997
ICU outcome	2.548	1.221	5.316	0.013

## Discussion

In this analytical cohort study, delirium was found to be prevalent in patients hospitalized for COVID-19 at KAMC; 13.5% (55 out of 406) of the patients treated for COVID-19 infection in the ICU developed delirium during their ICU stay. This is consistent with previous studies that have recognized an association between the development of delirium and COVID-19 in hospitalized patients worldwide [13-16].

Hospitalization due to COVID-19 is associated with worse outcomes. Recent studies have shown that delirium during the course of COVID-19 is associated with increased mortality and worse physical function after hospital discharge [17,18]. In a study conducted in the United Kingdom, delirium was reportedly prevalent and associated with poor functional outcomes in hospitalized patients [13]. A study conducted in São Paulo confirmed that delirium was associated with post-discharge functional and cognitive decline in COVID-19 patients [14].

In our study, delirium was significantly associated with older age, in accordance with a study in Italy that showed that delirium was associated with older age, neurologic comorbidities, including dementia and epilepsy, atypical symptoms of COVID-19, and worsening gas exchange at admission [15]. In addition, a study in Switzerland illustrated that delirium was a prevalent condition in older people admitted for COVID-19 and was linked to higher in-hospital mortality rates, with the main risk factor being pre-existing cognitive impairment [16]. Consequently, the findings of this study add to the growing body of work reporting the prevalence of and adverse outcomes associated with delirium in hospitalized COVID-19 patients. Our study also highlights the complications that delirium poses to cognitive abilities and mortality following the literature.

Additionally, our study showed that delirium was more prevalent in men (65.5%) than in women (34.5%). Several studies have reported that the male gender is an independent risk factor for developing delirium in hospitalized patients [19,20]. In addition, patients who developed delirium tended to be sicker and developed more severe infections, which has been shown to be a global trend.

Further studies are required to understand the mechanisms leading to delirium and its clinical and epidemiological outcomes. Although it is unclear whether any adverse consequences could be mitigated through better delirium care, the scale and potential for distress itself substantiate it as a high clinical priority.

This study has several limitations. First, patients with severe forms of COVID-19 may experience agitation as a direct consequence of breathlessness, high fever, and other signs of infection. If not correlated with altered consciousness and disorganized thinking, these manifestations do not represent delirium but require the administration of sedative drugs known to increase the risk of delirium [18]. Therefore, accurate delirium assessment in patients with severe COVID-19 is complex and challenging. Other limitations include the negative impact of being isolated from one’s support and family when infected with COVID-19 [12]. In addition, our analysis failed to assess the duration, severity, and temporal association of delirium with intensive care and other therapeutic measures. Lastly, it is noteworthy that our study was performed in a single center dedicated to high-complexity medical care, and our results should be interpreted with caution before being generalized to different populations.

Despite these limitations, nominal studies were conducted to investigate the link between COVID-19 hospitalization and developing delirium in the Kingdom of Saudi Arabia; hence, the significant results of this study pave the way for future research investigating the long-term impact of delirium and COVID-19, especially on Saudi nationals as they comprised the majority of our sample. In addition, we collected detailed clinical data from a large sample of patients, which provides new evidence that delirium could be considered to have a poor prognostic value for hospitalized patients with COVID-19 regarding ICU stay and death because it was associated with adverse outcomes. Our findings emphasize the need for dedicated early diagnosis and adequate management of delirium, which should be a central part of clinical protocols and mental health screening for the care of patients with moderate or severe forms of COVID-19.

## Conclusions

This study showed that age, nationality, comorbidities, infections other than COVID-19, and ICU outcomes were the main risk factors for delirium in hospitalized COVID-19 patients. These results highlight the importance of early findings on cognitive comorbidities and delirium in this population. The prompt recognition of delirium makes it difficult to ensure appropriate clinical care and prevent adverse outcomes in this population. Furthermore, as delirium is a preventable condition, it remains to be explored whether definite strategies targeting significant risk factors will positively impact its occurrence and outcomes after COVID-19.
